# Recovering the capability to work among patients with chronic low Back pain after a four-week, multidisciplinary biopsychosocial rehabilitation program: 18-month follow-up study

**DOI:** 10.1186/s12891-019-2831-6

**Published:** 2019-10-10

**Authors:** Maha E. Ibrahim, Kerstin Weber, Delphine S. Courvoisier, Stéphane Genevay

**Affiliations:** 1 Division of Rheumatology, University Hospitals of Geneva, Beau Séjour Hospital, CH-1205, 14 Geneva, Switzerland; 2 Department of Physical Medicine, Rheumatology and Rehabilitation, Suez Canal University, Ismailia, Egypt; 3 Medical Direction, University Hospitals of Geneva, Geneva, Switzerland; 4 Quality of Care Service, University Hospitals of Geneva, Geneva, Switzerland; 5 Department of General Internal Medicine, Rehabilitation and Geriatrics, University of Geneva, Geneva, Switzerland

**Keywords:** Return to work, Multidisciplinary rehabilitation, Chronic low Back pain, Work readiness

## Abstract

**Background:**

Chronic low back pain (LBP) is a leading cause of disability worldwide. Biopsychosocial rehabilitation programs have been advocated for its management, especially since the widespread acceptance of the biopsychosocial model of chronic pain. Despite extensive evidence of its short-term benefits, few studies have reported on its long-term effect and more specifically on indirect outcomes such as return to work and quality of life (QoL). The present study evaluated the long-term effect of a multidisciplinary biopsychosocial rehabilitation (MBR) program for patients with chronic LBP, for which short- and intermediate-term efficacy had been established, with an emphasis on recovering work capability.

**Methods:**

This prospective cohort study enrolled 201 patients on a four-week MBR program incorporating physical and occupational therapies and psychological counselling. Assessments occurred at program admission and discharge and at 6 and 18 months. Work capability, Oswestry Disability Index, Tampa Scale for Kinesiophobia, Core Outcome Measures Index (COMI), and Hospital Anxiety and Depression Scale were assessed. Multiple mixed models were used to detect changes in each outcome. Logistic regressions were calculated to identify predictors of recovery of work capability.

**Results:**

Of the 201 patients who fulfilled the eligibility criteria, 160 (79.8%) attended the discharge assessment, 127 (63.2%) attended the 6-month follow-up, and 107 (53.3%) continued to the 18-month follow-up. Initially, 128 patients (71.5%) had been on sick leave. At 6 and 18 months, 72 (56.7%) and 84 (78.5%) participants had recovered their work capability, respectively. There were significant improvements in pain, disability, kinesiophobia, and anxiety and depression scores over time. Patients who recovered work capability showed significantly greater improvements in their total COMI score, general QoL, and disability, which were the best three predictors of recovering work capability.

**Conclusions:**

This study extends previous results confirming the program’s contribution to recovering work capability among chronic LBP patients.

## Background

Chronic non-specific low back pain (LBP) mostly affects working populations and is a costly contemporary health problem in terms of absenteeism, lost productivity, substantial healthcare costs, and personal suffering [1–3]. LBP results in more years lived with disability than any other health condition and thus to significant disability insurance costs [3, 4]. Along with pain and impaired function, chronic LBP patients frequently experience anxiety, depression [5], and reduced quality of life (QoL) [6].

Patients reaching chronic stages of LBP seem to benefit from multidisciplinary approaches [7]. Multidisciplinary biopsychosocial rehabilitation (MBR) programs for LBP blend a variety of therapies administered by healthcare professionals from different specialties. They include physical therapy combined with psychological, social, and occupational therapies, and they aim to improve back-related physical function, address psychological issues, and target social or work-related behaviors [7, 8]. Several recent guidelines have advocated MBR for chronic back pain [9, 10].

In the absence of a universally accepted definition for *multidisciplinary rehabilitation*, different programs have used different intensities, durations, session designs, and patient selection criteria. This makes attributing the results of previous research to specific programs or populations difficult, especially as the literature presents more controversy than conclusiveness [11]. This lack of congruent results is especially true for indirect outcomes like return to work and QoL [12, 13]. However, analyzing these outcomes is important because MBR programs can be time-consuming, resource-intensive, and costly. Such outcomes should be key research considerations since they are primary determinants of the societal burden of chronic LBP—unfortunately, most studies do not report them [8]. Specifically, there is insufficient evidence about the effects of changes in outcomes such as pain, fear avoidance, depression, and anxiety on the evolution of work status [14, 15].

Since 2005, the University Hospitals of Geneva has been running an intensive, one-month MBR program for patients suffering from non-specific LBP and who have failed to attain an adequate response despite standard care from their general practitioner, including pain medication and individual physiotherapy [16–18]. This program was previously shown to be superior to a muscle reconditioning program proposed by the same institution, with the MBR program patients showing better improvements in disability and return to work [16]. However, that study was limited by a small sample size (45 patients) and a short follow-up (mean, 9 months). The present study’s objectives were, therefore, to assess whether the MBR program’s results remained valid for a larger cohort in the long-term (18 months) and to identify the factors which predicted recovering the capability to work among those patients.

## Materials and methods

### Participants

This prospective study’s cohort of 201 patients was enrolled at the University Hospitals of Geneva’s MBR program between 2006 and 2015. All eligible and willing patients attending either the Rheumatology or Rehabilitation clinics were invited to participate. Program inclusion criteria required French-speaking patients aged from 18 to 65 years old and suffering from non-specific LBP (with or without radiating leg pain) despite standard medical and exercise-based physical care prescribed by their general practitioner. Those in paid employment also had to be on sick leave. Exceptions were allowed in cases involving repetitive sick leave over the previous year or when patients presented with a high level of disability associated with significant psychosocial issues. Exclusion criteria were specific LBP due to infection, a tumor, spondyloarthropathy, radicular leg pain due to disc herniation, and neurogenic claudication related to spinal stenosis. Patients suffering from medical comorbidities which might interfere with or prohibit their participation (e.g., cardiac or pulmonary failure, severe mood disorder, disabling knee osteoarthritis) were also excluded, as were those with neck or diffuse chronic pain syndrome. Patients receiving disability benefit payments were likewise excluded. In Switzerland, sick leave can last for up to 720 days before a disability pension is allocated. All physical therapies outside the program were stopped for its duration as patients’ health insurance policies would not have reimbursed them. The relevant rheumatology or rehabilitation physicians adjusted pain medication on a case by case basis before program commencement.

### Treatment setting

The Geneva MBR program for chronic LBP was designed in 2005 to restore individuals’ musculoskeletal function. It includes significant cognitive–behavioral components and work-related goals and outcomes. Because of organizational constraints, the program only runs five times per year. A multidisciplinary team (a rheumatologist, a rehabilitation physician, a pain specialist, a psychiatrist, physiotherapists, occupational therapists, and a psychologist) followed small groups of four to six patients through the high-intensity, four-week, 100-h outpatient program, as described in detail in earlier publications [17, 18].

In summary, the program started with a one-day individual evaluation involving physical and psychological tests, and a clinical tool, based on a set of pictures, was used to gather important information with which to set meaningful, individual, therapeutic goals [19]. The tool identified patients’ priorities in their daily lives and their perceived degree of apprehension about performing those activities. This evaluation made it possible to negotiate personalized, realistic, meaningful objectives, which were both measurable and achievable in the care process. The goal-setting process was conducted during individual sessions supervised by the psychologist who had conducted the psychological tests. The psychologist did not provide any therapy during the program. The entire clinical evaluation and the goal-setting process were completed by the end of day two.

All sessions were group sessions, however, the physiotherapist had a panel of exercises to choose from and thus tailored the nature and intensity of physical exercises to each patient’s individual evaluation, physical capacities, psychosocial impairments, expectations, and priorities. Intensity was also adapted with regards to patients’ fear-avoidance beliefs and observed behaviors. Patients with a high level of fear-avoidance were initially prescribed lower objectives, but these were gradually raised when successfully reached. There were five types of group treatment sessions (Additional file 1). Type one involved 52 physical treatment sessions supervised by the physiotherapist, including cardiorespiratory fitness, muscular strength, muscular flexibility, stabilization exercises, relaxation, proprioception, and water aerobics. Type two involved 10 h of occupational therapy with an emphasis on physically difficult professional and daily life situations. These sessions addressed general needs (e.g., carrying, lifting, sitting, housekeeping tasks), individual patients’ goals for more specific tasks and, depending on the initial evaluation, additional workplace adaptations discussed individually with an occupational therapist. Most situations were dealt with during discussion sessions at the hospital but, in selected situations, on-site, workplace visits took place to improve the evaluation. Adaptations to workplaces were proposed according to specific needs; however, because of administrative constraints, some workplace interventions occurred after the program had ended. Thirdly, the rheumatologist or the rehabilitation physician led six patient-education sessions based on the non-injury [20] and biopsychosocial models. Fourthly, a weekly, one-hour support group discussion was led by a psychiatrist. Fifthly, 20 h of self-management exercises were scattered through the second half of the program.

The program aimed to increase muscle activity progressively and to help patients overcome their fear of movement whenever fear-avoidance beliefs were present. Patients’ kinesiophobia was estimated during their initial physical evaluations by observing their behavior or from their scores on the Tampa Scale for Kinesiophobia. As part of the program, patients were asked to develop personal booklets of illustrated, annotated exercises and techniques appropriate to their own condition. Throughout the program, they were encouraged to work out how they could integrate physical exercises into their daily life and possibly organize regular sporting activities. Each patient was explicitly expected to come to the last session with a detailed plan describing the what, when, where, and how of the exercise and sporting activities they would carry out over the following weeks, based on their experiences during the program. The MBR program was designed as an integrated program involving direct communication between team members and weekly multidisciplinary clinical meetings. Therapists shared objective and subjective clinical impressions during these meetings, also attended by the psychologist, and exercise requirements and individual goals were adapted to each patient’s progress.

Patients were informed that a multidisciplinary 3-h refresher course would be organized 5–6 months after the end of the program and that a postal survey would be organized at 18 months using the same patient-reported outcomes. The 6-month session’s main goal was to reinforce regular exercise and sporting activities. The program did not monitor patients’ adherence to their home-based programs, however, the refresher session motivated patients to continue following them and it repeated and reinforced messages about mobility, exercises, risks associated with fear-avoidance beliefs and behaviors, and the importance of goal setting for further improvement. An occupational therapist and a physiotherapist provided additional information according to individual needs. The occupational therapist helped facilitate returns to work during the months after the program, if necessary and possible, by promoting progressive returns to work with lighter duties. The planned postal survey was conducted at 18 months. In the absence of a response, a second questionnaire was sent, followed, if needed, by a telephone call.

### Outcome measures

At study entry, patients were categorized into one of four work statuses (working full-time, on sick leave, unemployed, or receiving social welfare payments) and this was recorded at every subsequent time-point to create a binary variable (had or had not recovered the capability to work). Overall capability to work was computed from patient-reported outcomes. The recovery of work capability was defined as either partial or full-time employment, being incorporated into an unemployment support program, and being on sick leave for a reason that had nothing to do with spinal pain.

The *Core Outcome Measures Index* (COMI) [21, 22] is a multidimensional, self-administered questionnaire that first assesses two pain symptoms (back and leg pain) on a 0–10 numerical rating scale. Five additional items assess the level of function, symptom-specific wellbeing, generic QoL, and work and social disability (averaged to form one disability score) over the previous month. These five items are rated on 5-point Likert scales with scores ranging from 0 (excellent condition) to 4 (worst condition). An overall score from 0 (best health status) to 10 (worst health status) can be calculated by averaging the subscales.

The *Oswestry Disability Index* (ODI) [23, 24] is the most commonly used, condition-specific outcome measure used in LBP management. Its 10 items, self-rated from 0 to 5, measure how back or leg pain affect the patient’s ability to manage everyday life. The sum of the 10 item-scores is multiplied by two so that the final score ranges from 0 to 100. A change of at least 10 points in the final range has been shown to be clinically meaningful.

The *Tampa Scale for Kinesiophobia* (TSK), developed by Miller et al. [25], is a self-administered measure of fear of movement. Kinesiophobia is defined as an excessive, irrational, and debilitating fear of physical movement and activity resulting from a feeling of vulnerability to painful injury or re-injury. Seventeen items are rated on 4-point Likert scales ranging from 1 (strongly disagree) to 4 (strongly agree). The total score thus ranges from 17 to 68, with higher scores indicating higher degrees of kinesiophobia.

The *Hospital Anxiety and Depression Scale* (HADS) [26] is a reliable, self-administered scale for detecting states of depression and anxiety in general hospital practice settings. It is composed of 14 items, each rated on a 4-point (0–3) response scale, with total scores ranging from 0 to 21 for anxiety and 0–21 for depression. A score of 8–10 is suggestive of the respective mood’s presence, and a score of 11 or higher indicates the probable presence of the mood disorder.

The *Medical Outcome Study Short-Form Health Survey* 12-items (MOS SF-12) [27–29]. Derived from the well-known SF-36, this generic, self-administered measure of subjective health-related QoL, looks at two distinct overall physical and mental health concepts known as the Physical Component Summary (PCS) and the Mental Component Summary (MCS). MOS SF-12 has a mean score of 50 and a standard deviation of 10: the higher the score, the better the QoL. It has demonstrated good psychometric qualities among LBP patients [30].

### Covariates

The following variables were included as covariates in the model: age, sex, and the duration of the incapability to work at baseline.

### Study design

Patients in this prospective cohort study completed all the questionnaire instruments on their admission to the Geneva MBR program, at the end of the 4-week program, and at the 6- and 18-month, one-day refresher courses. Failure to respond resulted in a second postal questionnaire being sent. Furthermore, at the 18-month follow-up, patients were telephoned to increase the response rate. All investigations were part of the treatment program’s routine clinical assessment. The study was conducted in accordance with the principals of the Declaration of Helsinki (2000 revision) and was approved by the local ethics committee.

### Statistical analysis

We used Fisher’s exact test for categorical variables and the Wilcoxon rank-sum test for continuous variables to compare the baseline characteristics of participants who were followed for 18 months with those lost to follow-up. To cope with those lost to follow-up, multiple mixed models were used for repeated measurements to assess significant changes to each outcome measure over time. An extreme-case sensitivity analysis compared study results with the best-case and worst-case scenarios. Finally, associations between patients’ clinical evolution and their recovered work capability were assessed using univariate and multivariate logistic regressions.

## Results

Of the original 201 patients who fulfilled the eligibility criteria, 160 (79.8%) attended through to the end-of-program follow-up, 127 (63.2%) attended the 6-month follow-up, and 107 (53.3%) continued to the study’s final 18-month follow-up. It is noteworthy that at the last follow-up, the postal address and telephone number of about 20% of study participants were out of date and no contact could be made. Regarding their socio-educational characteristics, slightly more patients were men, and most patients were in their forties, with a low-to-moderate level of education and employed in professions requiring physical effort (e.g., bricklayer, cleaner, nurse). At study entry, 128 patients (63.7%) were on sick leave and only 20 patients (10%) were still working. At baseline, the participants eventually lost to follow-up did not differ significantly from those who finished the study in terms of demographic, health, or occupational characteristics (Table 1). Furthermore, the extreme-case sensitivity analysis revealed no major differences between observed results and the best-case and worst-case scenarios, indicating that the study’s results were indeed robust.

**Table 1:** Baseline population characteristics (*n* = 201)

Characteristics		Continued to final follow-up (*n* = 107)	Lost to follow-up (*n* = 94)
Sex	Male	62 (57.9)	57 (60.6)
	Female	45 (42.1)	37 (39.4)
Age in years	M (SD)	41.01 (9.51)	38.8 (10.57)
Education	Primary	43 (40.2)	43 (45.7)
	Secondary	40 (37.4)	29 (30.9)
	University	13 (12.1)	16 (17)
	Not specified	11 (10.3)	6 (6.4)
Work status at baseline	Full-time job	10 (10.6)	10 (11.8)
	On sick leave	74 (78.7)	54 (63.5)
	Unemployed	3 (3.3)	8 (9.4)
	On social welfare	7 (7.4)	13 (15.3)
	Missing	13	9
Type of work activity at baseline	Intellectual	15 (15.6)	15 (16.9)
	Physical	52 (54.2)	54 (60.7)
	Both	29 (30.2)	20 (22.5)
	Missing	11	5
Duration of incapability to work at baseline	< 7 weeks	10 (9.3)	9 (9.6)
	7 weeks–3 months	10 (9.3)	6 (6.3)
	3–6 months	17 (15.9)	14 (14.8)
	6–18 months	32 (29.9)	31 (33.1)
	> 18 months	13 (12.1)	12 (12.8)
	Not applicable	25 (23.5)	22 (23.4)
Length of current incapability to work	< 7 weeks	6 (6.6)	4 (4.8)
	7 weeks–3 months	7 (7.8)	7 (8.4)
	3–6 months	12 (13.3)	6 (7.2)
	6–18 months	34 (37.8)	39 (47)
	> 18 months	31 (34.4)	27 (32.5)
	Missing	17	11
Health status at baseline	BMI, M (SD)	26.1 (4.15)	26.5 (5.6)
	Back surgery	20 (21.9)	18 (21.9)

At program discharge, after four weeks, only 39 of 160 patients (24%) were able to return to work. Interestingly, at 6-month follow-up, 72 of 127 patients (56%) had recovered their work capability, and at 18 months, 84 of 107 patients (78%) were capable of returning to work (Table 2).

**Table 2:** Course of outcome measures in patients with chronic LBP at end of program, 6- and 18-month follow-up

	Baseline (*n* = 201)	End of program (*n* = 160)	6-month (*n* = 127)	18-month (*n* = 107)	Coefficient (95% CI)	*p*-value^†^
Work status (recovered work capability, n (%)		39 (24.4%)	72 (56.7%)	84 (78.5%)		
COMI pain: M (SD)	6.2 (1.7)	5.7 (2.0)	4.9 (2.4)	4.9 (2.7)	−0.32 (−0.42; −0.23)	< 0.01**
COMI function: M (SD)	6.3 (2.1)	5.0 (2.3)	4.5 (2.6)	4.6 (3.0)	−.035 (− 0.47; − 0.25)	< 0.01**
COMI sQoL: M (SD)	8.6 (2.0)	7.4 (2.6)	6.5 (2.6)	6.1 (3.1)	−0.60 (− 0.72; − 0.48)	< 0.01**
COMI gQoL: M (SD)	5.8 (1.8)	5.0 (1.9)	5.0 (2.7)	4.6 (2.6)	−0.23 (− 0.34; − 0.14)	< 0.01**
COMI dis: M (SD)	6.4 (3.2)	5.0 (3.5)	3.8 (3.7)	3.1 (3.6)	−0.80 (− 0.96; − 0.63)	< 0.01**
COMI total: M (SD)	6.7 (1.5)	5.6 (1.9)	4.9 (2.3)	4.8 (2.6)	−0.44 (− 0.53; − 0.35)	< 0.01**
ODI disability: M (SD)	41.0 (13.3)	34.3 (16.2)	30.8 (17.8)	30.7 (19.8)	−2.15 (−2.76; −1.54)	< 0.01**
TSK: M (SD)	43.5 (7.7)	39.0 (8.8)	37.8 (9.2)	39.5 (10.3)	−0.97 (−1.35; − 0.58)	< 0.01**
HADS depression: M (SD)	8.7 (3.9)	6.5 (4.2)	6.9 (4.5)	6.3 (4.4)	−0.38 (− 0.55; − 0.21)	< 0.01**
HADS anxiety: M (SD)	9.8 (3.7)	8.5 (3.8)	8.7 (4.5)	8.5 (4.0)	−0.25 (− 0.40; − 0.10)	< 0.01**
SF-12 PCS: M (SD)	32.9 (7.6)	37.6 (9.1)	39.3 (10.8)	39.1 (11.6)	1.57 (1.06; 2.10)	< 0.01**
SF-12 MCS: M (SD)	38.3 (11.1)	46.2 (10.1)	43.1 (13.4)	41.6 (12.1)	0.66 (−0.01; 1.33)	0.051

*COMI* Core Outcome Measure Index, *sQol* symptom-related Quality of Life, *gQol* general Quality of Life, *ODI* Oswestry Disability Index (range 0–100), *TSK* Tampa Scale for Kinesiophobia (range 17–68), *HADS* Hospital Anxiety and Depression Scale (range 0–21), *SF-12* Medical Outcome Study Short-Form Health Survey, *PCS* physical quality of life (range 0–100), *MCS* mental quality of life (range 0–100)

^†^*P* values were obtained using multivariable linear mixed models

At baseline, the patients’ mean COMI pain score was 6.2 ± 1.7; the worst scores were in the domain of symptom-related QoL (8.6 ± 2.1). During the follow-up period, patients achieved significant reductions in all the COMI domains, exceeding minimum clinically significant improvements in the domains of disability and symptom-related QoL. A clinically significant reduction in the level of disability was also noted, with a mean decrease in the ODI of more than 10 points between baseline and 18-month follow-up (Table 2).

With regards to secondary outcomes, total TSK scores had decreased by the end of the program and at 6-month follow-up, but had risen again slightly at 18 months. Physical SF-12 QoL increased significantly throughout the study. Mean HADS depression scores had dropped below the clinically significant threshold (< 8) by the end of the four-week program, and they remained below it throughout the follow-up. On the contrary, mean HADS anxiety scores had fallen at the end of treatment, but never went below the threshold during follow-up. Mental QoL had increased by the end of treatment, but this rise did not last during follow-up.

Patients who had recovered their work capability at 18 months (84/107) had also had significantly better mean improvements at the end of the four-week program than those who had not recovered by the 18-month follow-up. These improvements were in total COMI score and in symptom-related and general QoL scores; improvement in the ODI was at the limit of significance (*p* = 0.051; Tables 3 and 4). After adjusting for demographic characteristics (age, sex, and duration of incapability to work), the variables able to predict recovery of work capability at 18 months were improvements in the total COMI score, in general QoL, and in the disability score as measured using the ODI (Table 3).

**Table 3:** Univariate and multivariate associations of the effects of interventions on outcome measures of capability to work at 18 months (recovered capability to work: yes, *n* = 84; no, *n* = 23)

Variables (T0–T1)	OR (95% CI)	Univariate *p*-value	Adjusted OR † (95% CI)	Multivariate *p*-value^‡^
COMI Total	1.54 (1.06; 2.38)	0.04*	1.61 (1.05; 2.66)	0.04*
COMI Pain	1.25 (0.95; 1.68)	0.11	1.25 (0.93; 1.73)	0.15
COMI Function	1.16 (0.93; 1.46)	0.18	1.25 (0.92; 1.71)	0.15
COMI Disability	0.96 (0.85; 1.12)	0.60	0.99 (0.84; 1.16)	0.91
COMI sQOL	1.29 (1.04; 1.64)	0.03*	1.27 (0.99; 1.70)	0.07
COMI gQOL	1.48 (1.11; 2.04)	0.01*	1.58 (1.12; 2.37)	0.02*
ODI	1.05 (1.01; 1.11)	0.051	1.10 (1.02; 1.18)	0.01*
TSK	1.05 (0.97; 1.12)	0.18	1.05 (0.98; 1.13)	0.19
HADS depression	1.12 (0.94; 1.32)	0.20	1.15 (0.95; 1.40)	0.15
HADS anxiety	1.20 (0.98; 1.48)	0.08	1.28 (1.01; 1.68)	0.055
PCS	0.96 (0.88; 1.02)	0.24	0.93 (0.84; 1.02)	0.15
MCS	0.97 (0.91; 1.01)	0.16	0.96 (0.90–; 1.01)	0.16

*T0* at baseline, *T1* end of program, *COMI* Core Outcome Measure Index, *sQol* symptom-related Quality of Life, *gQol* general Quality of Life, *ODI* Oswestry Disability Index (range 0–100), *TSK* Tampa Scale for Kinesiophobia (range 17–68); HADS, Hospital Anxiety and Depression Scale (range 0–21), *PCS* physical quality of life (range 0–100), *MCS* mental quality of life (range 0–100)

† Adjusted for age, sex, and duration of incapability to work at baseline

^*‡*^*P* values were obtained using univariate and multivariate logistic regression

**Table 4:** Univariate analyses of the effects of improvements in outcome measures at the end of the program on recovering the capability to work

Variables (T0–T1)	Recovered capability to work (*n* = 84) M (SD)	Did not recover capability to work (*n* = 23) M (SD)	*p*-value^†^
COMI Total	1.39 (1.59)	0.64 (0.85)	< 0.01**
COMI Pain	0.62 (2.03)	−0.09 (1.13)	0.04*
COMI Function	1.5 (2.31)	0.83 (1.20)	0.07
COMI Disability	1.36 (3.74)	1.79 (2.55)	0.53
COMI sQOL	1.93 (2.7)	0.52 (1.95)	< 0.01*
COMI gQOL	1.50 (1.53)	0.31 (1.53)	< 0.01*
ODI	9.2 (8.95)	4.5 (12.13)	0.09
TSK	5.21 (6.42)	2.92 (9.02)	0.26
HADS depression	2.52 (2.47)	1.67 (3.69)	0.29
HADS anxiety	1.90 (2.69)	0.79 (2.4)	0.06
PCS	−5.14 (8.11)	−2.62 (5.93)	0.17
MCS	−8.76 (11.3)	−4.22 (12.13)	0.18

*T0*, at baseline, *T1* end of program, *COMI* Core Outcome Measure Index, *sQol* symptom-related Quality of Life, *gQol* general Quality of Life, *ODI* Oswestry Disability Index (range 0–100), *TSK* Tampa Scale for Kinesiophobia (range 17–68), *HADS* Hospital Anxiety and Depression Scale (range 0–21), *PCS* physical quality of life (range 0–100), *MCS* mental quality of life (range 0–100)

† *P values were obtained using independent t-test*

## Discussion

The present study’s main goal was to assess the long-term outcomes of a multidisciplinary biopsychosocial rehabilitation (MBR) program on patients’ recovery of the work capability and on their pain, functional status, mood, QoL, and kinesiophobia. The cohort began with 128 patients (63.7%) unable to work because of LBP; they had low symptom-related QoL, a high level of disability, and above-threshold scores on scales measuring depression and anxiety. These results were not surprising because, in addition to disability and functional impairment, populations presenting with chronic pain have been shown to have more psychological comorbidities [5]. Furthermore, there is an established bidirectional relationship between psychological factors, particularly anxiety and depression, and chronic LBP [31, 32].

After the four-week MBR program, there was a substantial rise in work capability at the medium- and long-term follow-ups, together with improvements in pain, function, disability, general and symptom-related QoL, anxiety, and depression. At 18-month follow-up, 84 patients (78%) were either working or ready to return to work. Previous research, on a much smaller sample, had also shown a 78% return to work with a mean follow-up time of 8 months, and that study’s program was shown to be significantly superior to a standard muscle reconditioning program [16]. The present study confirmed and extended these results by demonstrating a consistent long-term improvement in recovery of work capability in LBP patients. A delayed return to work is known to be associated with high treatment and disability compensation costs [14]. Interventions which successfully target the return to work are highly valued since they address both the personal and societal costs of the disease. The present study supports existing data suggesting that access to multidisciplinary treatments is an important predictive factor of a progressive return to work following health issues or injury [33], particularly in cases of chronic LBP [34, 35].

Patients who recovered their work capability showed significantly greater improvements in total COMI scores and in general and symptom-related QoL. Total COMI, COMI QoL, and disability scores remained predictors of recovering work capability at 18 months after analyses were adjusted for patient characteristics such as age, sex, and duration of the incapability to work. These results are in line with previous research on chronic LBP patients, which found moderate evidence for negative associations between pain and function and between pain and return to work [14, 36]. The present results suggested that patients with a more holistic sense of well-being at the end of the four-week program were those more likely to return to work in the long run. They also suggested that the program’s individualized aspects—including personal goal-setting, targeting each patient’s particular difficulties in professional and daily-life situations, and tailored psychological support—could be important aspects to consider in future multidisciplinary programs aimed at improving patients’ work capability.

It is also worth noting that not all patients were capable of returning to work directly after the program. However, there was a steady increase in the number of patients who recovered their work capability during the follow-up, which confirmed the program’s long-term benefits. Although research has shown that return-to-work interventions are most successful when administered to patients whose incapability to work was shorter [37], this factor was not found to significantly affect return to work in our population.

The present study’s intervention also included workplace assessments, as it has been shown that these can have positive impacts on return to work [38, 39]. Although an assessment of working conditions was theoretically available for all participants, workplace interventions were conducted for less than 10% of them. Such interventions are not yet broadly accepted by employers in Switzerland, thus the contribution of workplace interventions to our overall results remains unproven.

The present study was also remarkable for the low response rate at follow-up, despite the significant efforts made to increase it. Although postal surveys are known for their lower response rates, we were more surprised by the low number of patients attending the 6-month follow-up session. During telephone calls, patients reported either having no time to participate (because of work constraints) or being dissatisfied that the program had not fully met their expectations. However, the study’s response rate (53.2%) was in line with current response rates observed in other studies [40]. Though there was a risk of attrition bias, this was mitigated by the fact that patients who were followed-up and patients who were lost to follow-up exhibited similar socio-demographic and disease characteristics. Nevertheless, this risk certainly limits the potential to generalize the study’s results.

Another significant limitation was the absence of a control group. This means that it is impossible to conclude that the program’s patients demonstrated a better clinical evolution than patients undergoing any other intervention. It also makes it difficult to refute the argument that the results could be attributable to LBP’s natural evolution. However, several previous conventional therapies had failed to adequately treat the patients included in the present program, some of which guidelines recommend as the standard care for this population [10]. We therefore believe that our results with this difficult patient population support the idea that the MBR program led to a positive clinical evolution. Indeed, a previous publication showed that, in the shorter term and with a similar population, this program was superior to an intensive physical rehabilitation program [16]. A third limitation is that the study did not record any pharmacological self-prescription or non-pharmacological self-management which patients might have carried out during the course of the study. Finally, the study lacked an associated cost-effectiveness study. Although the cost of the entire program per participant (about CHF 3600 or EUR 3240) may appear expensive, it is less than the recommended monthly minimum wage for the Geneva area (CHF 4000 or EUR 3600), suggesting that the program might have a favorable cost-effectiveness ratio.

Future studies should aim to determine which subgroups of the broad population of patients with chronic LBP should be referred to MBR programs. There is currently little information available on how to identify who might respond best to which treatments.

## Conclusions

Patients with chronic LBP who joined the University Hospitals of Geneva’s MBR program showed a significant increase in recovery of work capability, and this recovery extended up to 18 months after the program ended. Patients’ improvement in overall COMI, general QoL, and in functional capacity were the most important predictors of their recovery of the capability to work.

## Supplementary information

**Additional file 1:** Program Design.

## Data Availability

The datasets developed during the current study are available from the corresponding author upon reasonable request.

## Abbreviations

COMI: Core Outcome Measures Index
HADS: Hospital Anxiety and Depression Scale
LBP: Low back pain
MBR: Multidisciplinary biopsychosocial rehabilitation
MCS: Mental Component Summary
MOS SF-12: Medical Outcome Study Short-Form 12-items
ODI: Oswestry Disability Index
PCS: Physical Component Summary
QoL: Quality of Life
TSK: Tampa Scale for Kinesiophobia
